# Acknowledgement to Reviewers of the *Journal of Personalized Medicine* in 2013

**DOI:** 10.3390/jpm4010050

**Published:** 2014-02-27

**Authors:** Journal of Personalized Medicine Editorial Office

**Affiliations:** MDPI AG, Klybeckstrasse 64, CH-4057 Basel, Switzerland

The editors of the *Journal of Personalized Medicine* would like to express their sincere gratitude to the following reviewers for assessing manuscripts in 2013:

Alfirevic, Ana
Bartlett, Gillian
Bock, Christoph
Borry, Pascal
Bradbury, Angela
Brody, Lawrence
Byrne, Mitchell K.
Cadigan, Jean
Canestaro, William
Chen, Lei-Shih
Demner-Fushman, Dina
Diaz, Francisco J.
Dolbier, Christyn L.
El-Sohemy, Ahmed
Fahiminiya, Somayyeh
Forrest, Laura
Fratta, P.
Genovesi-Ebert, Federica
Georgiev, Asen
Graf, S
Grubs, Robin E.
Hall, Michael J.
Hornik, Chi Dang
Jani, Yogini H.
Jing, Xia
Joly, Yann
Joshi, Rohit
Joyce, Tony
Lam, Y. W. Francis
Lussier, Yves
Mcbride, Colleen
Mocanu, Stefan
Monia, Brett P.
Morris, Alan H.
Mossböck, Georg
Ossa, Diego
Overbey, Casey
Pai, Athma A.
Phillips, Kathryn
Pignato, Bill
Pillan, Margherita
Pirker, Robert
Pletcher, Mark J.
Powell, Charles
Quail, Michael
Ramon, Jan
Roberts, Robert
Rosell, Rafael
Sacristán, José A
Sargent, D.
Scalzo, Fabien
Schabath, Matthew B.
Schleidgen, Sebastian
Schröder, Annette
Schwartz, Stephen G.
Shuman, Cheryl
Stöger, Reinhard
Talameh, Jasmine
Tenenbaum, Jessica D.
Tomiak, E.
van de Poll, Matthijs
Vila, Joan
Voils, Corrine I
Voora, Deepak
Wasson, Katherine
Weeke, Peter
Williams, Marc
Yeo, Kiang-Teck J.

